# The Status of Older Adult Care in Contemporary Ghana: A Profile of Some Emerging Issues

**DOI:** 10.3389/fsoc.2019.00025

**Published:** 2019-04-11

**Authors:** Delali Adjoa Dovie

**Affiliations:** Department of Sociology, University of Ghana, Accra, Ghana

**Keywords:** old age, older adults, geriatric care, healthcare, institutional care homes, social interaction, loneliness, adjustment

## Abstract

The paper examines how the healthcare and social care pillars of social policy for aging societies shape inequalities in health and well-being at old age, utilizing qualitative and quantitative datasets. The results intimate the lack of geriatric infrastructure, hence the inadequacy of geriatric care provision for older adults. Systemic problems or gaps existent in Ghana led to private individuals taking advantage of the situation, turning it into an opportunity for service providers. Thus, the evolution of recreational/residential homes in Ghana is situated along three distinct patterns or forms namely the occasional, the adult day care center and residential archetypes. Collectively, these constitute formal and informal care facilities. These are often privately owned and at a cost. The nature of quality of care may be affected by the types of homes available, especially in the globalized cultural setting. A growing number of older adults resort to care homes as an alternative measure. These are discussed from two viewpoints. First, geriatric data generation, the absence of which impedes healthcare provision. Second, cash-for-care policies may exacerbate existing inequalities in care with negative consequences for health and well-being. In short, policies for aging populations are being implemented across Ghana with too little known about their consequences for inequalities in health and well-being in later life. The paper sought to address this knowledge gap by exploring a significant infrastructure by undertaking a systematic examination of how recent policy developments for aging exacerbate or reduce inequalities in health and well-being among older adults. The paper concludes that social policy for aging societies' specific key pillars (healthcare and social care research) offers opportunities for analyzing and understanding internal dynamics including the effects of policy implementation for inequalities in health and well-being at older ages, therefore enabling the identification of strategies to improve older adults' circumstances, without which older adult population will far outpace elder care provision.

## Introduction

### Role of Social Change

Social change has been the factor of transformation in contemporary life worldwide including Africa and Ghana. The latter is fostered by a myriad of drivers. Social change is induced by modernization, urbanization, globalization (Apt, 2012; Kpessa-Whyte, 2018). This, in turn, has necessitated societal and familial changes, which could be explained by social and economic conditions. The social conditions encompass weakened extended family support system (Aboderin, 2006; Doh et al., 2014; van der Geest, 2016; Dovie, 2018a); inadequate formal support infrastructure (Aboderin, 2006; Dovie, 2018a) and increasing nucleation of the family. Similarly, the neglect of older people (Dovie et al., 2018) may be responsible for the lack of care for them. The economic conditions consist of the occurrence of “brain drain” among doctors and care staff who migrate to developed countries, labor and economic conditions that force women, who are often made responsible for the care of the family to work outside the home (Schatz and Seeley, 2015; van der Geest, 2016; Coe, 2017) among others. All these have implications for geriatric and institutional care particularly for older adults.

### Concerns With Care Provision for Older People

Care for older people is a widespread phenomenon. In some high-income countries, there is variable funding for social care; for example, Germany has a special tax to help fund this. In the United Kingdom (UK), social care for patients in institutions is means tested, for example, above a certain level of assets this is self-funded. The same is in the United States. Consequently, this is an issue facing all countries (Robertson et al., 2014). The evolution of the aging population is faster to live in low-and-middle-income countries (LMICs). Eighty percent of older adults will live in LMICs (Agbogidi and Azodo, 2009; WHO, 2017a), including Ghana.

A study conducted in Malaysia observed that older people with education, income and the need for healthcare was associated with healthcare services utilization (Yunus et al., 2017). Hamiduzzaman et al. (2016) also ascertained factors that influenced older female's utilization of healthcare services in rural Bangladesh. They identified deprivation of adequate education, social and economic dependency on males and family, inadequate and ineffectual institutional healthcare arrangements and misappropriation of funds. In a similar study, Hren et al. (2015) revealed that older persons in Slovenia often sought out-patient-department services and hospitalization. The study found that age and education were the factors that influenced the use of healthcare and other related services. Also, worth reiterating is the fact that increase in the population of older adults is occurring along the same timelines as the breakdown in the traditional system of social protection and care (Kpessa-Whyte, 2018) due to urbanization, socio-economic development, and globalization. This has implications for the well-being of older adults and policy.

In sub-Saharan Africa (SSA), a rapidly aging population is presenting challenges to healthcare systems. Doctors need specialized knowledge to be prepared for the increase in age-related medical conditions. Further, older adults are likely to be challenged with physical, mental, and social changes that require adjustment as they attain later life (Martin et al., 2008). These are indicative of an impending burden of elder care. Geriatric care is associated with hospitalization irrespective of the form it takes. A study conducted by Frost et al. (2015) shows that 4% of medical schools in SSA taught geriatrics while 40% had no geriatrics teaching. Additionally, the most significant perceived barriers to geriatric education and the attendant care were a lack of staff expertise (72%), lack of funding (52%), and absence of geriatrics in the national curricula (48%). There are still a large number of medical schools in SSA who do not teach geriatrics. Healthcare needs such as orthostatic hypertension measurement/management, vision assessment, toileting schedule (Colon-Emeric et al., 2018; Sharma et al., 2018) may require comorbidity management. This has implications for the quality of care delivered (Colon-Emeric et al., 2018) to them.

Also, substantial social class differences exist in preferences for activities including perceptions about institutional care. It also has economic effects on these range of possible activities. Some social class differences in activities culminate from differences in what people are taught to prefer whereas others reflect differences in financial capacity (Atchley and Barusch, 2004) notwithstanding the notion of need. The divisions between social classes are becoming wider, not narrower. In the UK, for example, figures from the Equality Trust (2017 cited in Manstead, 2018) show that the top one-fifth of households have 40% of national income, whereas the bottom one-fifth have just 8% (Manstead, 2018). Research by Savage et al. revealed that the differences between the social classes they identified extended beyond differences in financial circumstances. There were also marked differences in social and cultural capital, as indexed by the size of social networks and the extent of engagement with different (cultural) activities, respectively.

### The Institutional Care Challenge

Populations around the world are aging rapidly, and this demographic transition is placing new demands on societies to provide comprehensive systems for long-term care at home, in communities or institutions. An institutional home denotes a place of residence for older adults who require continual nursing care and have significant difficulty in relation to coping with the essential activities of daily living (ADLs). In sub-Saharan Africa, 46 million older people live in the region and this number is expected to more than triple (to 165 million1) by 2050. A significant proportion of these people will require long-term care at some point in their lives (De-Graft Aikins and Apt, 2016).

Long-term care can be provided in a range of settings including people's homes and by a range of people. Currently, families provide the most long-term care in sub-Saharan Africa and do so without any organized training or support. Reliance on families alone to provide this care culminates in inconsistent care quality and places a particularly heavy burden on women and girls. Moreover, it may be unsustainable given the rapidly increasing number of older people (WHO, 2017a, p. 1). WHO's (2017b) “Global Strategy and Action Plan on Aging and Health” (1) calls on all countries to develop a system of long-term care. The Strategy stresses that no single system of long-term care can be applied in every setting, even in countries with similar resource constraints. Governments need to take into account the number of older people as well as their need for long-term care, existing models of service delivery, the availability, and skills of unpaid caregivers.

Demographic trends including slowed fertility, the killing of the middle generation by HIV/AIDS and an aging population have led to a “care deficit” for the young and old. Those who fill this care deficit are often women, and in many cases, older women (Schatz and Seeley, 2015). As reported elsewhere, 60% of AIDS orphans in Zimbabwe, South Africa, and Namibia live with grandparents (Apt, 2012). The majority of South African HIV caregivers in one study were female (68%); of these, 23% were 60+ (Steinberg et al., 2002). Therefore, age and gender come together to shape the experience of care.

Gender assists in the definition of how care is framed, who provides it and how it is experienced. In other words, gender determines who gives care and who receives the same (Schatz and Seeley, 2015). In East and Southern Africa, both men and women live with impacts of the care deficit. However, sexual division of labor has meant women predominantly fill the void in care (Oppong, 2006). Cultural beliefs about “maternal instinct” and men's “natural” roles as breadwinners have led to assumptions that women are better suited to the daily care of the young, old, and sick, while men are supposed to provide financial assistance.

For example, women (and men) may see care for an aging or sick husband as a continuation of “wifely duties.” Likewise, caring for the young or sick may not be reported as care work, as it fulfills routine family expectations and obligations (Schatz and Seeley, 2015). On the other hand, men's physical and emotional care work is often more visible. Because men's care work is further outside men's normative familial roles, it is more likely to be perceived as care work and reported as such.

The Westernization and/or modernization of our society has resulted in the challenge of caring for older people. With the increasing rate of population aging and increased life expectancy, the need for institutional homes may be required as a supportive measure. The upsurge in need for an institutional support system for older adults in Ghana is becoming imperative due to their inability to care for themselves, loneliness due to the loss of family relations including social change, the lack of spouses and children, modernization, urbanization, migration, multiple careers including busy work schedules. These factors have all made care for older people an impending challenge. Older people take up residence in institutions. This implies that social care even in a number of Western countries is means tested, which is a key issue going forward (e.g., responsible for a considerable amount of bed blocking in UK hospitals) (Robertson et al., 2014), whereas in developing countries such as Ghana, it is not means tested as yet, perhaps because state institutional homes are non-existent. Besides, older adults requiring care are still looked after within the informal structure of the family (Apt, 2012) in some LMICs.

There is also an increase in the number of neglected, abused, and abandoned older adults. Further, the modern economic system has projected old age as a social problem. In developed countries, when older people become handicapped by virtue of illness and disability, they take up residence in institutional homes. However, such facilities are not extensively provided in developing countries. As a result, older people are cared for at home. The situation may be different in the near future, necessitating taking refuge in institutional homes. These homes may be classified as formal and informal, which may draw on paid and unpaid labor (Daly et al., 2015).

Relocation adjustment in nursing homes Lee (2010) purports predictors such as self-efficacy, self-reported health, preconception about nursing homes, emotional support from staff and other residents, family satisfaction and general satisfaction with the facility in question. Nursing homes are a relatively new phenomenon in the Ghanaian context. People do not cope or adjust to aging or old age in isolation. Instead, they do so in the company of others who provide social and emotional support. This may depend on the financial independence and sacrificial investments in their children, who may become adults with the moral duty to support their aged parents (Doh et al., 2014; Dovie, 2018a). This implies that prior lifestyle determines to a great extent the quality of care and support older adults are accorded, and when it becomes inevitable for them to depend on others.

There exists universal healthcare (such as in South Africa and countries such as Namibia), where elements are free such as in government/faith hospitals and facilities, and the extent of co-pay afterward (Ataguba and Akabili, 2010). Ghana operates the National Health Insurance Scheme (NHIS) to provide citizens with health insurance. The level of premiums citizens must pay varies according to their level of income. Elements are free particularly for people aged 70+ (Dovie, 2018b, in press), which is a reflection of discrimination even among older adult members. Also, Ghana still has a high burden of infectious diseases and a very growing burden of non-communicable diseases (NCDs) including hypertension, diabetes, and stroke (De-Graft Aikins and Apt, 2016) that must also be tackled within available resources. In other words, “the NHIS prescribes the same basic healthcare without taking into consideration the tertiary healthcare needs of older people especially in the area of non-communicable diseases, such as retention of urine, incontinence, prostrate and colon cancers” (van der Geest, 2016, p. 11).

De-Graft Aikins et al. (2016) posit that research on aging in Ghana has focused on six empirical areas: demographic profiles and patterns of aging; the health status (physical, mental, and sexual) of older adults; care and support for older adults; roles and responsibilities of older adults; social representations of aging and social responses to older adults; and socio-economic status, social and financial protection and other forms of support for older adults. This study falls within the care and support for older adults' framework. Yet, few investigations or little work has been done on geriatric care and institutional homes and adjustment to it in Ghana. Hence, the paper seeks to address the following research questions: How can geriatric care be improved on to ensure adequate related care in Ghana? What has been the evolutionary pattern of institutional homes in Ghana? To what extent do older adults adjust to old age by living in institutional homes? The first and second questions were addressed using qualitative interview data while the third was answered with a combination of quantitative and qualitative data. It is noteworthy that for this paper, institutional home, and nursing homes are used interchangeably.

## Materials and Methods

The research project began with some concerns and questions about older adults and care system in Ghana. The study used quantitative and qualitative datasets and a cross-sectional design to investigate the healthcare and social care pillars of aging social policy and how these shape inequalities in health and well-being. Use was made of in-depth interviews to explore geriatric care dynamics, while the questionnaire survey provided the necessary data for the development of an understanding of Ghana's repertoire of institutional care homes including qualitative interview data obtained from medical doctors, nurses, near old workers, and retirees.

### Site Selection

Accra and Tema were chosen as the study sites because they are typical of major African cities that are privy to extended family support system and associated issues, hence their selection. They were also chosen because they depict the epitome of an urban setting which articulates the deepened prongs of individualization and the reality of the weakened nature of the extended family support mechanism. Also, they present a web of social relations, occupational diversity, a variety of activities as well as various events over time that provides richer and more interesting data (Ghanaweb, 2015), and suitability.

### Sample Selection and Recruitment

The study adopted the simple random sampling technique in selecting the respondents. This technique of sampling was used in this research project for two reasons. First, almost old and older adults are a distinct group of study participants. Second, the study purports to identify particular types of cases for in-depth investigation (Neuman, 2004). For the quantitative data, 230 workers in the near old age category and retirees took part in the study. In the case of the qualitative data, 16 in-depth interviews were conducted with a section of individuals with the requisite information. The sampling process entailed the random sampling of working individuals aged 50–59 years (50) in the Tema Metropolis based on a list obtained from Tema Development Corporation. People aged 60+ years (180) were selected using a list obtained from the National Pensioners' Association in Accra and Tema. The samples were selected from a total population of 364 and 700+ respectively. The sample size was calculated using the following formula: *n* = 2(Za+Z1–β)^2σ2^/Δ^2^, with a power of 80% and constant of 1.65 and a *p* < 0.05 (Kadam and Bhalerao, 2010).

Therefore, 270 questionnaires were given out, and 230 were returned. Although the sample size was constrained by resources, 230 observations were selected as adequate for the study. The sample is large enough to help address the research questions accurately. The study also sought to explore the association between living in institutional homes and adjustment to old age based on sex. The usage of the simple random sampling technique means the results are statistically representative and to the general population. Thus, generalizability to the general population is permissible.

### Research Instruments

#### Questionnaire

A questionnaire containing three sections was used in data collection. Section one was on the socio-demographic characteristics namely age, educational level, and ethnicity. The second section explored issues of geriatric concern. Section three comprised social care dynamics were measured with 5-point verbalized scale from “‘extremely associated” to “not at all associated.” The questionnaire was created based on previous research, input from colleagues and also the study's research interests. Examples of questions that have been previously used in published studies include questions about the perceived older adult care (i.e., relational needs, care homes, and geriatric training) (Frost et al., 2015; WHO, 2017a; Dovie et al., 2018). After the initial pool of questionnaire was written, qualified experts were made to review it, especially for grammatical corrections and accuracy. Before conducting a pilot of the questionnaire on the intended respondents, it was tested on a small sample of 30 individuals following the guidelines of Perneger et al. (2015). Afterward, a pilot test among the intended respondents for initials validation was undertaken. All participants completed the same questionnaire.

Together these were collectively contextualized to fit this study and the Ghanaian scenario. The survey questionnaire instrument's reliability was ensured in diverse ways, namely, facilitation by clear instructions and wording of questions. The questionnaire contained standardized instructions namely “please tick where appropriate.” Also, trait sources of error were minimized through interviewing respondents at their convenience. To attain this, interview appointments were scheduled severally. The validity of the survey data was attained following Nardi's (2006) guidelines. The validity of the data was obtained from face-to-face interviews. Also, the survey sought an alternative source for confirmation through further in-depth interviews.

The administration of the questionnaire took the form of face-to-face interviews including self-administration. The face-to-face interviews were conducted in both English language and Ghanaian languages namely Ga, Ewe, and Twi.

#### Interviews

The sample for the qualitative phase was selected from that used in the quantitative phase as well as other stakeholders—physicians, nurses, social workers, etc. utilizing the purposive sampling technique. Purposive sampling was used for diverse reasons including its importance in the selection of participants who had specific characteristics such as sources of information. The 16 participants were divided into the three planned interviews based on their convenience (i.e., participants' preference of time and location). Participants were excluded if they were younger than 50 years. The interviews were conducted in February 2017, ~2 months after completion of the questionnaire. The interview themes that emerged were related to the perception of geriatric care training, the emergence of institutional care homes, and suggestions for future studies.

The interviews were designed to gain an understanding of older adults' perceptions regarding geriatric care and social care dimensions of aging policy. The interviews focused on geriatric care and aspects of institutional care. The interviews lasted ~45 min. Initially, the researcher reminded participants about the aim of the study and that the discussion would be used to suggest future directions.

Each in-depth interview took the form of a semi-structured interview and was conducted individually in the participant's office or chosen place. The interviews were audio-taped. Face-to-face interviews are endowed with the merit of providing pertinent information while allowing the researcher the opportunity to control the line of questioning (Neuman, 2004).

#### Data Analysis

Methodological triangulation was deployed to include the combination of methods to understand and explain (Greenstein et al., 2003) geriatric and institutional care. The answered questionnaire were cleaned and serialized for easy identification. The survey data were entered into Statistical Package for Social Science (SPSS) and were analyzed with selected descriptive statistics namely frequencies, percentages, Chi-square statistics, and Cramer's V test.

Transcripts from the interviews were subjected to thematic analysis. Thematic analysis entails the process of encoding qualitative as well as textual information. Despite the strict procedural nature of coding and themes that emerged from constant immersion with qualitative data, Joffe and Yardley (2004) contend that thematic analysis is more exploratory. For the interviews, data analysis was first conducted by the researcher and subsequently by an independent researcher with experience in qualitative data analysis to increase confirmability and dependability. Both researchers ensured dependability by keeping a coding manual, which entailed original extracts from the interviews and definitions of the emergent themes (Johnstone, 2006). Inductive thematic analysis using NVivo10 software was undertaken (Bazeley and Jackson, 2014). Each of the researchers read the scripts in detail, and then individually coded and categorized data from the same interview. Data from the interviews were coded by the researchers and across the entire interview data capturing diverse views. Through comparison, constant refining resulted in a list of themes (e.g., geriatric care challenges, steps in comprehensive geriatric assessment, archetypes of institutional homes in Ghana, institutional residence for older adults, adjustment to old age homes, the aging policy) with their importance determined by frequency, multiplicity of participants' views as well as uniqueness.

## Results

### Socio-Demographic Characteristics of Respondents

The study population consisted of males (47.3%) and females (52.8%) aged between 50+ years (Table 1). The respondents (58%) were married. Most of the respondents had some level of education. On the whole, the highest educational level attained by the majority of the respondents (80.3%) was tertiary education. The discussion above shows that the sample is composed of high proportions of university graduates. Some of them are engaged in paid work (51.3%).

**Table 1:** Socio-demographics.

**Variables**	**Characteristics**	**Percentages(%)**
Age	50–59	23.9
	60–69	23.9
	70–79	23.9
	80–89	23.9
	90+	4.3
Sex	Male	47.3
	Female	52.8
Education	No-formal	9.2
	Pre-tertiary	17.5
	Tertiary	80.3
Marriage	Married	58.0
	Divorced	23.5
	Widowed	7.5
	Single	11.0
Occupation	Working	51.3
	Not working	48.7

It is worthy of note that the reference universe of the sample is significantly restricted to urban, educated older people.

### Qualitative Data: Geriatric Care Dynamism in Ghana

#### Geriatric Care Challenges

The interview data reflects the myriad of challenges regarding geriatric care for older adults in Ghana encountered from the viewpoint of the services provided to them, such as limited data available concerning health services for older people; the lack of geriatricians or oriented physicians; there are no preferential services in most health facilities; limited staff training in the field of geriatric care including unaccompanied patients often neglected on admission. This challenge may be deeply rooted in relational issues. In terms of training and research, the qualitative data shows that a few educational institutions namely the Ghana College of Physicians and Surgeons offer geriatric fellowship programs. The University of Ghana and University of Cape Coast also have curriculums in geriatrics including undergraduate curriculum in gerontology. The biggest challenge in all these is the lack of political will to pass the aging policy bill into law. Interestingly, this very bill and/or law began its process of enactment in 2003 but has not yet attained its logical conclusion.

The intensity of older adult care challenge has been highlighted by the quote below:

Health services provision for older adults is an issue of increasing concern, especially in industrialized nations. High rates of institutionalization in older adults' population are attributed to the lack of a comprehensive assessment of the medical, social, functional, and psychological needs of high-risk groups (health personnel).

It is in the light of this that comprehensive geriatric assessment (CGA) is imperative as discussed below.

#### Comprehensive Geriatric Assessment

The CGA is a systematic approach to collecting data with health status evaluation. It also entails the integration of the functional and medical goals of care in order to improve clinical outcomes as well as patient satisfaction. From the functional dimension, it has been observed that health maintenance in this context cannot be underestimated since aging is associated with the increasing burden of chronic diseases or conditions among other health challenges. These are known as the “geriatric giants comprising incontinence, immobility, instability and nutrition” (physician 1). It is because of this that “falls in old age homes are more prevalent” (nurse 1).

An interview with a fellow shows that “the Ghana College of Physicians and Surgeons has a home care and palliative care vehicle donated by the church of Pentecost” (fellow), which when functional may facilitate care provision for older adults. Another quote intimates that: “educating the caregivers is important in the case of depression, for example” (paramedic1). This shows how caregivers care for older adults as well as manage themselves.

The geriatric care system has specific prerogative requirements and/or goals for both older adults and physicians. On the one hand, older adults and their families are to regain lost function and maintain independence. On the other hand, those of the physicians encompass the diagnosis and treatment of acute medical illnesses as well as the management of underlying chronic diseases.

From a functional perspective, CGA findings and/or analysis are integrated with the patient's functional capacity. It also allows physicians to among other things optimize healthcare outcomes, dependent on multidisciplinary team constituted by the care team, social workers, geriatric nurses, trained nurses, psychologists, chiefs, pastors, pharmacists including physicians.

#### Steps in Comprehensive Geriatric Assessment

Four distinct steps in CGA pertain, namely medical history; assessment; physical examination and laboratory; care plan or domain. The first step in CGA entails the taking of medical history–presenting complaints provided by the patient, family members, caregivers, or wards. It also involves geriatric reviews of and screening for fall risks, incontinence, abuse, spirituality, memory, depression, sleep, and a host of others. Collectively, these have outcomes for biomedical and social data. The biomedical data comprises nutritional and past medical history, medications, duration of use and adverse drugs. The social dimension of data entails the individual older adult's social status, family support system and documentation of advanced directive such as leaving a will. The second step which relates to the assessment exercise revolves around sleeping nature, diagnosis of spiritual distress, the need for blood transfusion, which some belief system(s) does not support, and a host of others.

The third step comprises physical examination and laboratory, expressed in terms of general examination and visual acuity, heart, breast examination, cardiopulmonary, abdominal, lower body as well as neurologic. The care plan domain relates to geriatric syndromes namely dementia, falls, incontinence, and general geriatric diagnoses such as hypertension, pneumonia, and contextual diagnosis. Medicals pertain to drug inspection; nutrition—continence, defecation, cognition, emotion, mobility; cooperation with care plan and caregivers pertain. This has implications for health maintenance in its entirety. Therefore, it is worth noting that the health maintenance dimension speaks to issues of preventive geriatrics.

#### Archetypes of Institutional Homes in Ghana

Ghana's (private) institutional home care sector only began to emerge over a decade ago. The interview data shows that the market for this has been created by a myriad of factors namely increase in the population of older adults, increased life expectancy, changing disease patterns, changing family structures as well as inadequate public provision of institutional homes.

Presently, the evolutionary pattern of institutional homes indicates that it exists in three distinct archetypes. First, the occasional archetype which takes the form of a rare phenomenon entails the bringing together of older adults to a social gathering by a lead individual, where they are feted and socially interacted with. It serves as a means of reducing boredom and loneliness, albeit for a short while. It is a form of respite particularly for those who have no one to depend on in terms of social interaction. This archetype of institutional homes does not, however, involve the housing of older adults in a residential facility. Further, quite apart from fostering social engagement, it also fosters the social integration of older people. This form of care may exist alongside the residential or institutional home version of care which seems to be more holistic, sedentary and regular. An example of this can be found “in Ho in the Volta Region, under the auspices of the current Moderator of the Evangelical Presbyterian, Church, executed by delegated individuals. This has been on-going for about 3 years” (retiree 1).

Social interaction with older adults gives them a sense of being a part of the general society while averting stereotypic perceptions about older people. This kind of perception is quite illusive because old age is an inevitable stage such as retirement which every human being who does not die prematurely is bound to experience, perhaps differently. It is not an experience for a select group of people but for all and sundry.

Second, the adult daycare center archetype, which according to the retirees pertains to daycare centers wherein older people visit a given day care center, where they eat and do everything else except sleeping there overnight. In this context, older adults eat, interact with other facility users as well as play games such as ludo, cards and a host of others during the day and then depart to their respective homes at the end of the day. This archetype more extensively keeps older adults busy and away from boredom and loneliness in the same sense as archetype one including keeping them abreast with issues trending in their societies and other societies in general and issues of older people in particular. Thus, keeping them engaged and active with implications for successful aging. This exerts an external influence on older adults while facilitating their adjustment process. The Henri Dei Recreational Center located at Osu in the Greater Accra Region of Ghana is a typical example of this archetype. HelpAge Ghana also operates a day center for older people 60+. The services it provides comprise healthcare and health screening, recreation, meals (lunch) as well as handicraft training.

Third, all the participants intimated that the residential archetype denotes a real institutional home. It provides both non-medical or social and medical or skilled care including meals. By illustration, it provides residential facilities namely beds for overnight stay overs, social and recreational activities, medical care and/or health professionals (e.g., nurses, doctors, paramedics) as well as social workers. However, the provision of such services by the existing residential archetype is not for free. Instead, mostly patronage is at a fee ranging between 500.00 and 1,500.00 Ghana cedis ($104.84–$315.52) per month per head. This category of nursing homes is presently privately owned in Ghana and tailored to the affordability of the affluent in the Ghanaian society. Currently, it is patronized by parents and/or older family relations of the affluent who appear not to have the time to care for the former personally. Most of these beneficiaries suffer from dementia, mental disorders of diverse forms and a host of others, and who could not be handled within the ordinary family home contexts including not having to employ professionals to care for them in their ordinary family contexts. They may be localized mostly in Accra and Tema as it is in the case of Mercy Mission or have branches across the country as exemplified by CarePlus Ghana.

Generally, institutional care homes are characterized by the process of social engagement and social integration. The occasional archetype is more informal whereas the adult-day care archetype is more formal, although it could be informal in some cases. The first and second archetypes may be said to be in their early forms mostly because they do not provide residential facilities and are therefore not characterized by these features. They do not provide medical care except for social care. This depicts an evolutionary pattern of institutional care homes from a simpler form to a more sophisticated one which the residential archetype depicts. The first two archetypes provide not-for-profit home-like care to older adults. Currently, in Ghana, the residential archetype is formal, yet widely non-governmentally owned. These existing archetypes of institutional homes seek to herald the inception and institutionalization of nursing homes nationwide in Ghana including the need for it irrespective of the acceptance of its existence in the Ghanaian society. This indirectly calls on the state for the need to institute “state-owned” institutional care homes for older people across all the 10 regions of the country. This has become imperative, particularly because of the cost involved in terms of accessing private homes including accessibility broadly to the majority of older people beyond the affluent in the Ghanaian society. Similarly, the affluent may be coerced to patronize state-owned homes dependent on their availability. Significantly, the establishment of state-owned institutional homes will infuse equity into the mode of accessibility to such homes and the varieties thereof.

There is the likelihood that the family members of older people who relocate to institutional homes may not visit them at all, culminating in older people being entirely abandoned. The success of institutional homes may be affected by cultural underpinnings. These issues have been articulated as follows:

We Africans, we have several and different cultural backgrounds. First, over there they do not stay in families which is very different for us. Second, to get people to work there, it might require people who are very devoted because fancy a home which has 30 or 40 people, come together and live in family homes. However, we ask ourselves whether we can do it here? This is the reason why I feel that at the moment, it is because of our culture and the way we are living here. But over there by their culture, they live alone, and I am wondering whether even family members go to visit them. I am wondering, I do not know. So here, it might be good doing it, but then we might start from a point with people who are devoted to this just course. I know people may go there and may be abandoned halfway, and it will be worse. The only thing is that let us train our children and grandchildren well so that they can know our values well so that they can treat older people well before they die (retiree 2).

This may imply that the family tie is the most important factor for the well-being of older adults. It also seeks to establish a preference for traditional methods of being cared for by the younger generation as well as family members.

The statement below vividly confirms the opposing perspective to adjustment in old age. This validates some of the challenges that may be associated with living in institutional homes. This has been demonstrated by the statement below:

I wonder if someone can adjust to leaving home to staying in nursing homes, whether that is feasible in our society. Because if you leave your home and move into someone else's house, what you will hear from others and people who are close to you and who talk directly to you will even kill you before your time. This is with regard to the particular change effected. So, to me, I do not think there is a link between these two whatsoever. They are in fact two different things all together. They are two extreme issues. Stay in the family, if you have a good family. In some places, when you stay in your home, and then they cater for you at old age, thank God. If you do not take care, if you are expected to die at 85 years, you will die at 102. But here frustrations, nobody will visit you when you are in an old age home. Do you know loneliness is what kills some people most? At home, even it happens, but at least you will have one or two people to come and visit you. But over there as soon as they know you are there, they would not even come to visit you. You are entirely forgotten. They may even make your funeral for you while you are still alive (retiree 3).

The participants indicated that older adults do not have to live in institutional homes because they could be easily forgotten. Moreover, instead of that, they could even die from loneliness. It is observed that while older adults live in their own homes, they seem to have been forgotten, let alone having relocated to an institutional home. In other words, the injurious effects may far outweigh the positive notion of adjustment. This is because it may pre-dispose its inmates to further neglect. Additionally, these homes must be supported with resources to optimize their functionality. In consequence, it has been argued that:

There are plenty of examples. You may live in Osu and say I am going to Teshie to be with my cousin. Everybody at Osu will become happy because you are going. When you return, the only thing they will ask you is, so soon you have returned? You see, it is because of the economy. I always say it (retiree 4).

In effect, it is intimated that:

Out there in these institutional homes, people come to support some of these places, and the government also comes in to support. In fact, people donate as we have in children's homes when children are sent there, even though the parents are alive, they dump them there and forget about them entirely. This is the reason why I say; there could be further neglect (retiree 4).

#### Quantitative Data: Institutional Residence for Older Adults

**Figure 1:**
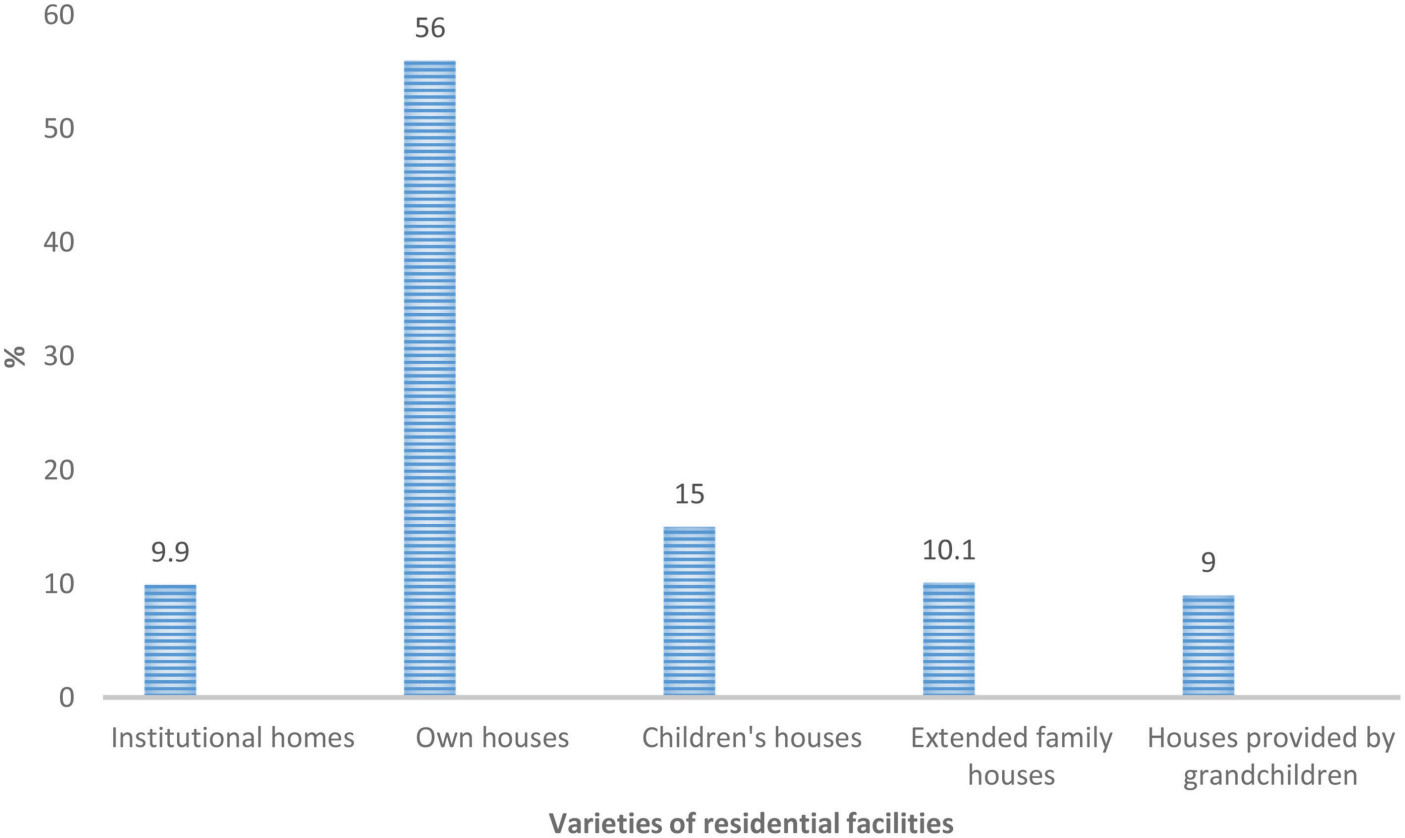
Residential facility options to older adults.

**Figure 2:**
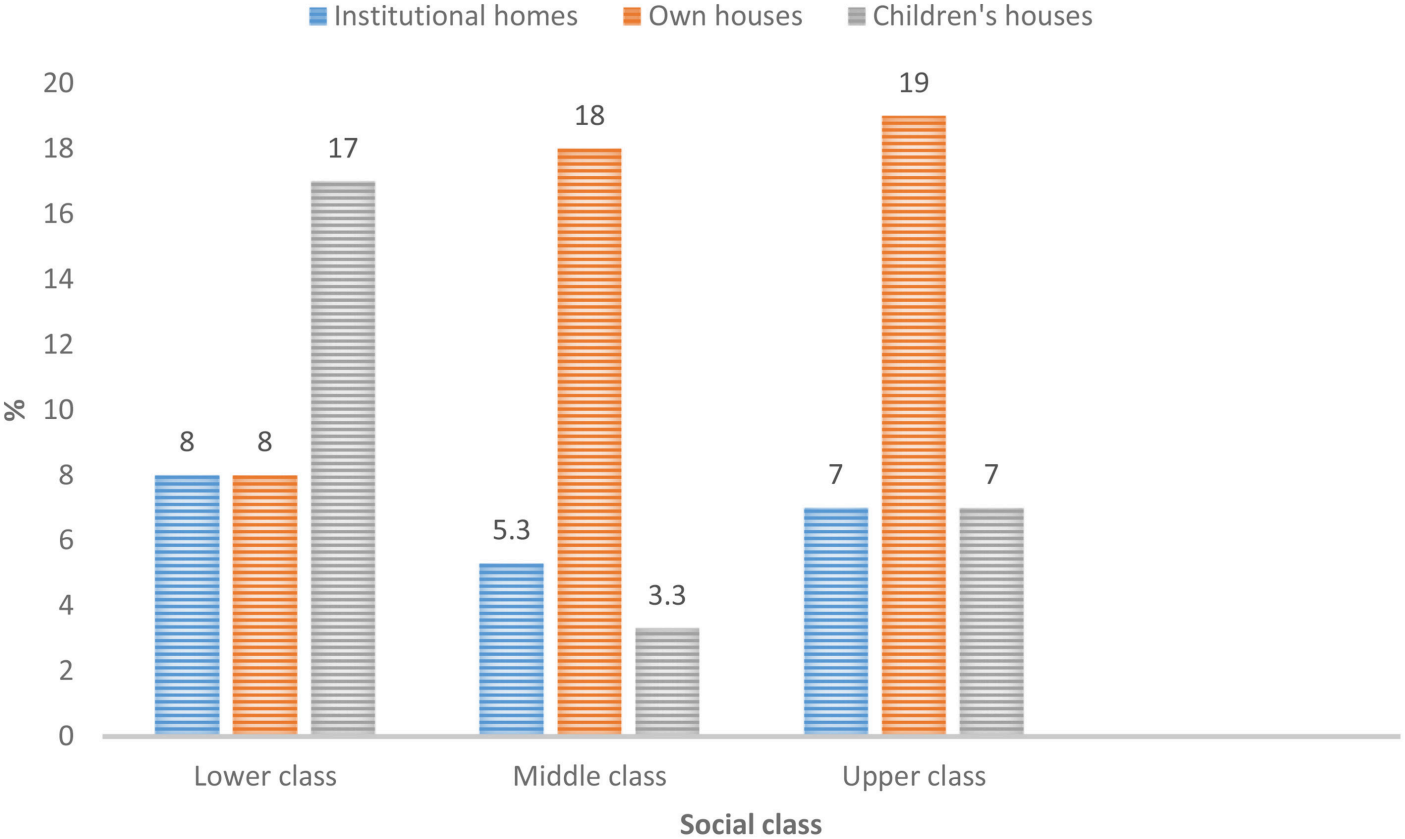
Class-based perception of residential facility patterns for older people.

There are a variety of residential facilities for older adults that may serve as their places of abode namely institutional homes, own houses, children's houses, extended family houses, houses provided by grandchildren including where ever older adults choose to reside. Out of these, own house (56%) was indicated by the majority of the sample studied (Figure 1). Interestingly, the latter option also implicitly refers to all the options mentioned before it. Indicating institutional homes in the above residential facility list may suggest that older adults have the propensity to reside in the institutional home when the need arises. This gives credence to the acknowledgment of the effects of the forces of social change and/or modernization. The results establish how social class shapes the respondents' perceptions of institutional care. In consequence, many of the respondents within the middle and upper classes outlined own houses as an option for residential facilities. All the classes articulated the fact that the options older people may adopt is institutional homes, whereas more lower-class respondents preferred living with children (see Figure 2 for details). This implies that whereas the middle class proffers institutional care as an option, the lower class looks forward to living with children. The social class thus facilitates alignment with regard to existing changing modes of residence for older adults. In other words, perceptions of social care are influenced by social class.

#### The Linkage Between Living in Old Age Homes and Adjustment to Old Age

The results intimate that institutional care homes although a new phenomenon in Ghana tends to facilitate adjustment to old age discussed in the section below. Hence, there are two distinct perspectives to this termed adjustment and non-adjustment perspectives. The adjustment perspective demonstrates that not having anyone to care for older adults suggests the need for institutional homes. In assessing the linkage between living in institutional homes and adjustment to post-retirement life, the quantitative data show by the majority of the respondents (57.3%) that institutional homes facilitate adjustment to post-retirement life (see Table 2 for details). This is because, in institutional homes, older people may feel and have a sense of belonging in the midst of their fellows. It may assist in cutting off stress, boredom, and loneliness. Further, many older adults really do not have relations they go back to after retirement, particularly, the childless ones including those who are neglected. These may be well taken care of, loved as well as the ability to socialize and interact with peers. Older adults may also obtain assistance from or with the support of caregivers in institutional homes enabling them to adjust to life including the conditions in which they may find themselves. Finally, older adults can understand themselves better when they live together.

**Table 2:** Do institutional care homes facilitate adjustment?

**Responses**	**Percentage (%)**
Institutional care homes facilitate adjustment	132 (57.3)
Institutional care homes institutional care homes do not facilitate adjustment	98 (42.6)
Total	230 (100)

The Cramer's V test value of 0.57 (Table 3), establishes that there is a relatively strong statistically significant relationship between living in institutional homes and adjustment to post-retirement life.

**Table 3:** Association between living in institutional homes and adjustment to old age based on sex.

**Value**	**Degree of freedom**	**Asymptotic significance (2-sided)**
Pearson chi-square	75.03	3.052
Cramer's V test	0.57	1.490
N of valid cases	131	

Perhaps, the reason why woman are more inclined (than man) to think institutional homes are good for adjustment to old age is that men and women are affected differently—older women are more likely to say they feel lonely than older men.

The non-adjustment perspective opposes the above-indicated adjustment assertion. Thus, ~23.0% of the respondents do not believe there is any linkage between living in nursing homes and adjustment to old age. Instead, it is believed that the little pension money is spent in institutional homes, instead of older adults having houses of their own after retirement. Besides, people in society may think that the inmates of institutional homes did not work hard enough to be able to acquire decent accommodation for themselves in life. Circumstances beyond the control of the individual involved may necessitate living outside an acquired house, the expenses of which may be paid by significant others including their children. The preceding perception marks a blatant rejection of the residential home model.

#### Aging Policy

The voices articulate the fact that “Ghana is quick to sign on to international declarations, for example, 60 years in Ghana, 65–67 years in the west” (near old participant 1). This is interspersed with policy gaps in service provision and laws for older adults. The absence of a structured and consolidated policy environment exacerbates inequalities in older adults' healthcare and well-being. This is reminiscent of a serious omission of older adults' issues in distinctive policy/law instruments. Presently, there is no single policy instrument dwelling on the unique issues related to older adults in its entirety. Policies that address older people's issues are in bits and pieces or scattered across a range of others including the 1992 Constitution, whereas education, children, and youth have concrete policies or laws designated to them. This notwithstanding, the country's population is increasingly aging. This trend depicts a grievous omission of the plight of older adults. The extent of seriousness is here expressed in the following quote: “The sin of commission is as grievous as the sin of commission” (social worker 1). Last but not least, it is worth reiterating the fact that, “attitudinal change is the key thing that is needed for improved aged care going forward” (near old participant 2).

## Discussion

This paper presents findings of a mixed-methods study in relation to care issues affecting the Ghanaian older person, in which the results of quantitative and qualitative analyses shed light on perceptions on two dimensions of care discussed in this paper, namely geriatric and institutional care homes.

### The Geriatric Care Concerns

Geriatric care is associated with hospitalization, bringing to the fore the fact that older adults have a myriad of problems with their living conditions such as the absence of segregated care for older persons at health facilities including healthcare expenditures (Dovie, 2018b; Sharma et al., 2018; Ghiara and Russo, 2019). Constraints regarding geriatric care and social services and care have culminated in increased needs for formal income support including care provision for older adults. Also, changes occurring in the older population will further challenge this and other existing arrangements.

### The Essence of Institutional Home Care

Institutional homes seem to be a relatively new concept comparatively in the Ghanaian society including some parts of Africa. This may be due to the rate of population aging including the increasing decline in the extended family system as a mechanism of social support, busy work schedules, childlessness, and the loss of children. In effect, a (non-formal) care industry with workers from the labor market has gradually emerged in response to the dynamics of social change. The emergence of which can be found in three distinct forms such as the occasional, the daycare center and residential archetypes. However, as van der Geest (2016) found a reliable overview of the number and quality of such setups is non-existent.

The quantitative findings show that living in institutional homes correlates positively with adjustment to preretirement life. This is an indication that nursing homes are a life support place for older adults when they have no other option for living in any other place. This is a depiction of adjustment to living in institutional care homes. Adjustment to old age may take diverse forms including material adjustment, social adjustment, and emotional adjustment to home environments, all of which are essential. As a result, it is suggested that institutional homes could initially be made a home care institution for older adults who have no one to care for them, particularly, those who are childless or neglected. Later, this trend could be followed by other categories of older adults. The key concern, however, pertains to quality of care in residential homes.

This study revealed two distinct features. First, Western culture espouses living alone during younger years, while coming together to live in community or family homes. Second, they seem to have the requisite caliber of workers in institutional homes. Since the phenomenon is a new concept in the Ghanaian context, the requisite caliber of workers with the dedication and devotion may be non-existent. This can be developed through training since the need for such caliber of workers is essential. The preceding paragraph emphasizes the fact that while institutional homes may facilitate adjustment to old age, they may also signify houses for the poor or forgotten people

However, there exists a disconnection between the care plan for multifactorial problems including falls and actual resident care. Thus, periodic checks by the Ministry of Gender Children and Social Protection designated staff may help in streamlining such situations. This also signifies the need for state instituted and managed nursing homes nationwide.

Opposed to the adjustment to institutional care homes as indicated earlier, is the perceptions that older people are incapable of adjusting to nursing homes. In other words, contrarily, the non-adjustment perspective espouses the reverse, stressing the myriad of challenges that inmates of the institutional home may encounter. The preceding perception marks a blatant rejection of the residential home model. This is in line with what van der Geest (2016) found that handing over the responsibility to outsiders or professionals in institutions, as has become a common practice in many “Western” countries, is widely rejected in Ghana. The findings from the survey corroborate those from the qualitative data on the issue. In consequence, institutionalized care is regarded as an unfortunate development in the Ghanaian society (van der Geest, 2009). As Van der Geest et al. (2004) wrote, a similar situation occurred among migrant populations in the Netherlands, who once detested nursing homes.

In essence, the study shows that the breakdown in the traditional pillars of social support for older people is paving the way for newer appreciations to elder care provisioning expressed in the emergence of institutional care homes in contemporary Ghana.

### Limitation of the Study

The limitation of the study merits discussion. The study failed to investigate adjustment using a nursing home adjustment scale and older people in its entirety as study participants.

### The Way Forward

The results of the study have implications for aging social policy. First, long-standing gaps in knowledge about geriatric health needs exist, while a rapidly aging population is presenting challenges to healthcare systems. In preparing for the increase in age-related medical conditions, medical doctors require specialized knowledge. Community health nurses should be used to provide similar services such as those provided by maternal healthcare and immunization for older adults. Aging experts need to advocate the teaching of geriatrics and gerontology in medical schools nationwide.

Second, the most significant perceived barriers to geriatric care were a lack of staff expertise, lack of funding, and absence of geriatrics in the national curricula. Improvements in geriatric education and care should be implemented through local approaches and national policy while appreciating the cultural context, and particularly economic constraints to prepare future doctors for the increasing challenges of an aging population.

Third, medical records can be abstracted by trained physicians, nurses and other medical staff supplemented with a review of facility logs. Fourth, a clinical vignette containing components such as environmental modifications, exercise/rehabilitation, and psychoactive medication reduction may also go a long way to facilitate the quality of geriatric care delivered by hospitals and care homes and received by older adults. These are to be completed by personnel at different levels—as a facility level measure of care quality. Besides, vignettes seem superior to abstractions especially regarding measuring nursing home quality. When undertaken, it is imperative that the process measures be validated before their adoption by regulators or administrators widely.

Fifth, the structuring and ordering of healthcare services to older adults needs to be regimented along the following lines: segregation of older adults' folders from those of the younger groups and treatment should not follow the first come first served modality but with consideration for older adults. Better still, health facilities need to consider creating separate clinics for older adults. The passage of the national aging policy bill into law is imperative. These go a long way to ensure optimal aging among older adults. Future research may explore the completion of vignettes by the staff most engaged in care provision in nursing home settings. This includes seeking to distinguish between differences in the quality of care between nursing units within the same facility to enable the targeting of nursing improvement efforts to those with lower performance.

Sixth, it is paramount to consider these distinct perspectives and categories for the implementation of effective intervention programs in relation to adjustment to old age in Ghana. Seventh, the caring functions of the family must be strengthened using the provision of resources in support of care for older adults. Eighth, factors of relocation, residence, social support, and facilities should be taken into account with regard to enabling older adults to achieve adjustment to institutional homes in general and old age in particular. Ninth, the government through the Ministry of Gender Children and Social Protection in collaboration with other stakeholders must institute state-owned institutional homes nationwide, which may serve the purpose of contributing to a wide variety of care for older adults at large. Perhaps, it will be worth having a guest house facility as part of such homes where relatives and friends of inmates may visit and stay for a couple of days. This is particularly essential for emotional release and psychological stability. Finally, studies must investigate adjustment using an institutional home adjustment scale.

On the whole, the absence of a structured and consolidated policy environment exacerbates inequalities in older adults' health care and well-being. Ghana's health system is not responsive to the healthcare needs of older adults. Policies in Ghana concentrate more on children, youth and education, hence the key challenge is the “factoring in” of older adults into social policy (healthcare and social care) to ensure some level of balance in the policy domain, albeit within the frame of severe resource constraints. As Lloyd-Sherlock (2002) argues, this may require external donors, non-governmental organizations including the state to undertake priority re-orientation for the onward accommodation of eldercare exigencies. This suggests that adequate state finance of old age support is a feasible policy option. In furtherance to this, its attainment entails the mainstreaming of older adults into primary healthcare in its entirety and social care provision in a composite manner to ensure that the extent of resources and attention they receive is appreciable. This has implications for chronic health conditions including NCDs in later life.

## Ethics Statement

The University of Ghana's Institutional Review Board approved the project. Confidentiality and anonymity were ensured.

## Author Contributions

The author confirms being the sole contributor of this work and has approved it for publication.

### Conflict of Interest Statement

The author declares that the research was conducted in the absence of any commercial or financial relationships that could be construed as a potential conflict of interest.
